# Fetal functional imaging portrays heterogeneous development of emerging human brain networks

**DOI:** 10.3389/fnhum.2014.00852

**Published:** 2014-10-22

**Authors:** András Jakab, Ernst Schwartz, Gregor Kasprian, Gerlinde M. Gruber, Daniela Prayer, Veronika Schöpf, Georg Langs

**Affiliations:** ^1^Computational Imaging Research Lab, Department of Biomedical Imaging and Image-guided Therapy, Medical University of ViennaVienna, Austria; ^2^Division for Neuroradiology and Musculoskeletal Radiology, Department of Biomedical Imaging and Image-guided Therapy, Medical University of ViennaVienna, Austria; ^3^Department of Systematic Anatomy, Center for Anatomy and Cell Biology, Medical University of ViennaVienna, Austria; ^4^Computer Science and Artificial Intelligence Lab, Massachusetts Institute of TechnologyCambridge, MA, USA

**Keywords:** fetal functional MRI, fetal brain connectivity, prenatal development, connectome, fetal brain development

## Abstract

The functional connectivity architecture of the adult human brain enables complex cognitive processes, and exhibits a remarkably complex structure shared across individuals. We are only beginning to understand its heterogeneous structure, ranging from a strongly hierarchical organization in sensorimotor areas to widely distributed networks in areas such as the parieto-frontal cortex. Our study relied on the functional magnetic resonance imaging (fMRI) data of 32 fetuses with no detectable morphological abnormalities. After adapting functional magnetic resonance acquisition, motion correction, and nuisance signal reduction procedures of resting-state functional data analysis to fetuses, we extracted neural activity information for major cortical and subcortical structures. Resting fMRI networks were observed for increasing regional functional connectivity from 21st to 38th gestational weeks (GWs) with a network-based statistical inference approach. The overall connectivity network, short range, and interhemispheric connections showed sigmoid expansion curve peaking at the 26–29 GW. In contrast, long-range connections exhibited linear increase with no periods of peaking development. Region-specific increase of functional signal synchrony followed a sequence of occipital (peak: 24.8 GW), temporal (peak: 26 GW), frontal (peak: 26.4 GW), and parietal expansion (peak: 27.5 GW). We successfully adapted functional neuroimaging and image post-processing approaches to correlate macroscopical scale activations in the fetal brain with gestational age. This *in vivo* study reflects the fact that the mid-fetal period hosts events that cause the architecture of the brain circuitry to mature, which presumably manifests in increasing strength of intra- and interhemispheric functional macro connectivity.

## INTRODUCTION

Human brain development is a finely orchestrated process that relies on the sequential execution of genetic programs, complex cellular interactions, and the formation and resolution of transient organs (Price et al., 2006; Kostovic and Judas, 2010). The protracted telencephalon maturation and the evolutionary expansion of the neocortex are prerequisites for our intellectual capacity (Clancy et al., 2001; Preuss, 2009), and many highly developed cognitive abilities are linked to features that are unique to humans (Bystron et al., 2008; Fjell et al., 2013). Early brain development encompasses the rapid growth of the size of the cerebral cortex and the increase in its cellular variability, associated with the expansion of transient fetal structures, including the subventricular zone and the subplate (Molnár et al., 2006; Rakic, 2009). When compared to the developmental trajectories of great apes, the growth rate of the human brain diverges during mid-gestation, and further expansion of white matter volume is observed in early postnatal life (Rilling, 2014). Specific areas, such as the association areas of the neocortex and areas related to language processing, exhibit exceptionally large subplate zones in humans (Rakic, 2009), and these differences are also mirrored in the cortical expansion during childhood and protracted myelination, which is completed in adulthood (Geschwind, 1964; Hill et al., 2010).

In contrast to our knowledge on brain circuit formation and histogenesis from post mortem studies and functional examinations in early childhood (Johnson, 2001; Price et al., 2006; Huang et al., 2009; Judas et al., 2013), to date, we have only a limited understanding of emerging neural activity during human fetal development. The complex connectional architecture of neural ensembles is a fundamental characteristic of the adult brain, and we are beginning to understand how its variability across individuals is linked to cognitive ability (van den Heuvel et al., 2009), or neuropsychiatric disorders (Greicius, 2008). Some functional networks in the fronto-parietal and ventrolateral frontal cortex seem to be specific to humans (Mantini et al., 2013; Neubert et al., 2014), and the functional variability across the adult cortex correlates with cortical expansion from the primate to human brain (Mueller et al., 2013). To understand normal fetal functional brain development, it is of vital importance to identify critical periods of developmental vulnerability in which harmful environmental agents can manifest as a deterioration of mental capability or cause psychiatric diseases (Rice and Barone, 2000). In these phases of interest, functional assessment of the fetal nervous system would open a novel window on prenatal diagnostics and prognostics (Jakob and Beckmann, 1986; Paul et al., 2007; Schlotz and Phillips, 2009).

Studying fetal behavior with ultrasound has been adopted as a marker for neural development in clinical practice, offering an indirect view of the underlying functional processes (Pugash et al., 2008; Morokuma et al., 2013). MRI in the fetal brain has become increasingly feasible and clinically important due to its higher tissue resolution and better visualization of normal and pathological development of macroscopic anatomy or white matter microstructure and connections (Glenn and Barkovich, 2006; Kasprian et al., 2008; Jokhi and Whitby, 2011). Advanced imaging techniques, such as spectroscopy, serve as indicators of naturally changing metabolite levels during gestation (Kok et al., 2001), and characterize impaired development caused by primary brain malformation, hypoxic-ischemic injury, infection, or a combination of those factors (Mailath-Pokorny et al., 2012). BOLD fMRI has probed macroscopic-scale intrinsic neural activations across the entire life span (Buckner et al., 2013), and has an unsurpassed role in recent population-wide neuroimaging endeavors (Nooner et al., 2012; Uğurbil et al., 2013; Yan et al., 2013). Initial fetal investigation of spontaneous, intrinsic functional activity and functional brain connectivity has established intra-lobar activations (Schöpf et al., 2012) and cross-hemispheric connections in normally developing fetuses (Thomason et al., 2013).

In this paper, we provide a comprehensive view of the developing functional networks in the fetal brain. We capture this process by examining changes in the whole-brain functional connectivity architecture (Achard et al., 2006; Rubinov and Sporns, 2010; Zalesky et al., 2010) during gestation. We measured thalamo-cortical, cortico-cortical, intra-hemispheric, and inter-hemispheric functional connections, and organized them into a whole-brain graph (Fransson, 2005; Bullmore and Sporns, 2009). The graph-based method (Rubinov and Sporns, 2010) detected regional and temporal heterogeneity of functional connectivity development across the cortex before birth, reflecting the possible sequence of maturation in cortical areas.

## MATERIALS AND METHODS

### ETHICS AND SAFETY OF FETAL MRI ACQUISITIONS

Based on the follow-up studies of infants or children who had been exposed to MR imaging *in utero*, no gross abnormality, disease, or disability likely to be related to MR exposure could be demonstrated (Kok et al., 2004). The study protocol was approved by the local ethics committee, the mothers gave written, informed consent prior to the examination, and research was conducted according to the principles expressed in the Declaration of Helsinki.

### STUDY POPULATION

This cross-sectional study included thirty-two singleton fetuses (16 female, 16 male) between GWs 21–37 (mean: 29.2; SD: 4.9) with normal brain development. The distribution of the gestational ages was the following. Second trimester (<26th GW): eight fetuses, early third trimester (26–29th GW): eight fetuses, 29–37th GW: 16 fetuses. Between May 2010 and August 2012, fMRI was performed for 200 fetuses. From of this cohort 141 scans were excluded due to image artifacts, uncontrollable fetal or maternal motion. After retaining the technically successful scans, the next exclusion criterion was the presence of brain abnormalities. Brain abnormalities were confirmed with the use of structural MRI in 27 of the cases. Therefore, 32 scans were retained for further analysis, which had no confirmed brain abnormalities. In the remaining study cohort, the indication for the mothers to undergo fetal MRI included: gastrointestinal pathology (two subjects); maternal clinical conditions (nine subjects); micrognathia or clefts (three subjects); renal and urogenital pathology (six subjects); thoracic pathology (one subject); asymmetry of the lateral ventricles (LVs; six subjects); and pathologies of the diaphragm (five subjects). Our final investigations were restricted to the 32 subjects who were proven negative for these suspected anomalies.

### IMAGE ACQUISITION

Functional magnetic resonance imaging was carried out on a 1.5 T clinical scanner (Philips Medical Systems, Best, Netherlands) using a sensitivity encoding (SENSE) cardiac coil with five elements, utilizing single-shot gradient-recalled echo-planar imaging (EPI). The pregnant women were examined in the supine or left decubitus position (feet first), and no contrast agents or sedatives were administered. The MRI scans were acquired between May 2010 and August 2012. All investigations were scheduled between 7 and 9 am. Image matrix size was 144 × 144, with an FOV of 250 mm × 250 mm, a TE/TR of 50/1000 ms, and a flip angle of 90°. The resting-state scan comprised 50 image volumes with slices obtained perpendicular to the fetal brainstem, 10–30 slices were acquired and scan time was 1 min. The fMRI examinations followed the diagnostic (e.g., T2-weighted anatomical) scans, utilizing institutional protocols similar to those of previous reports (Schöpf et al., 2012).

### IMAGE PROCESSING

**FIGURE 1:**
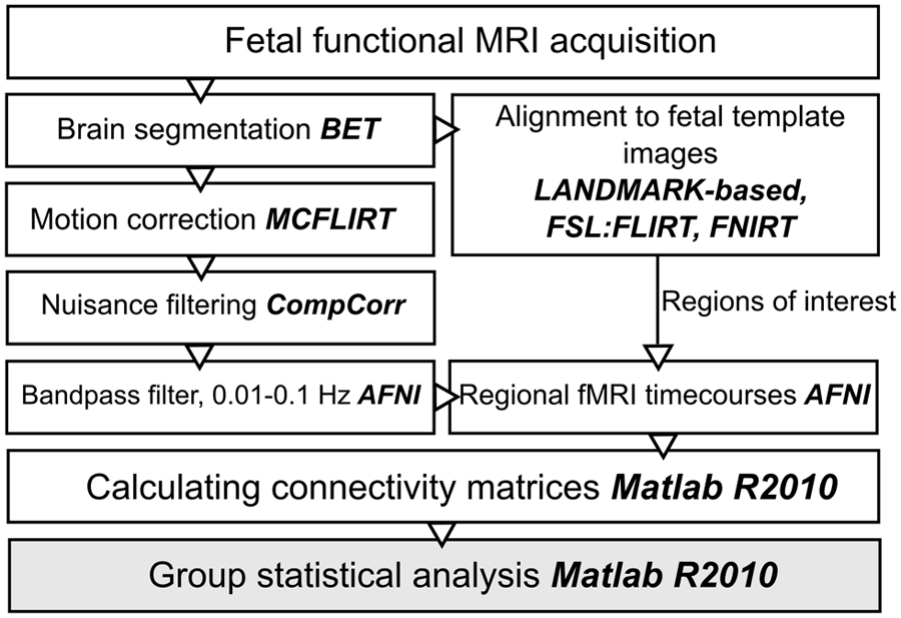
**Work diagram of the fMRI processing steps.** The names of the respective image processing toolboxes are given in bold. BET, brain extraction tool; FLIRT, FSL linear registration tool; FNIRT, FSL non-linear registration tool; CompCor, component based noise correction method; AFNI, analysis of functional neuroimages.

The next paragraphs introduce an optimized work-flow for processing fetal functional images, which required effort in the direction of adapting conventional toolboxes and further extending them by fetal-specific developments. An overview of post-processing steps is given in **Figure 1**.

### SPATIAL NORMALIZATION OF FUNCTIONAL MR IMAGES TO GESTATIONAL WEEK-SPECIFIC BRAIN TEMPLATES

A practical way to interrogate the developing fetal neuroanatomy is to pool image datasets of the study cohort according to GW and use templates that depict the mean anatomical configuration of each GW. During the spatial normalization, we used the reference template images provided by Rueckert (2012) which are high-resolution representations of the changing fetal brain from the 23rd to the 37th GW. Template creation procedure described in: (Serag et al., 2012), while the fetal templates were accessed from Rueckert (2012).

**FIGURE 2:**
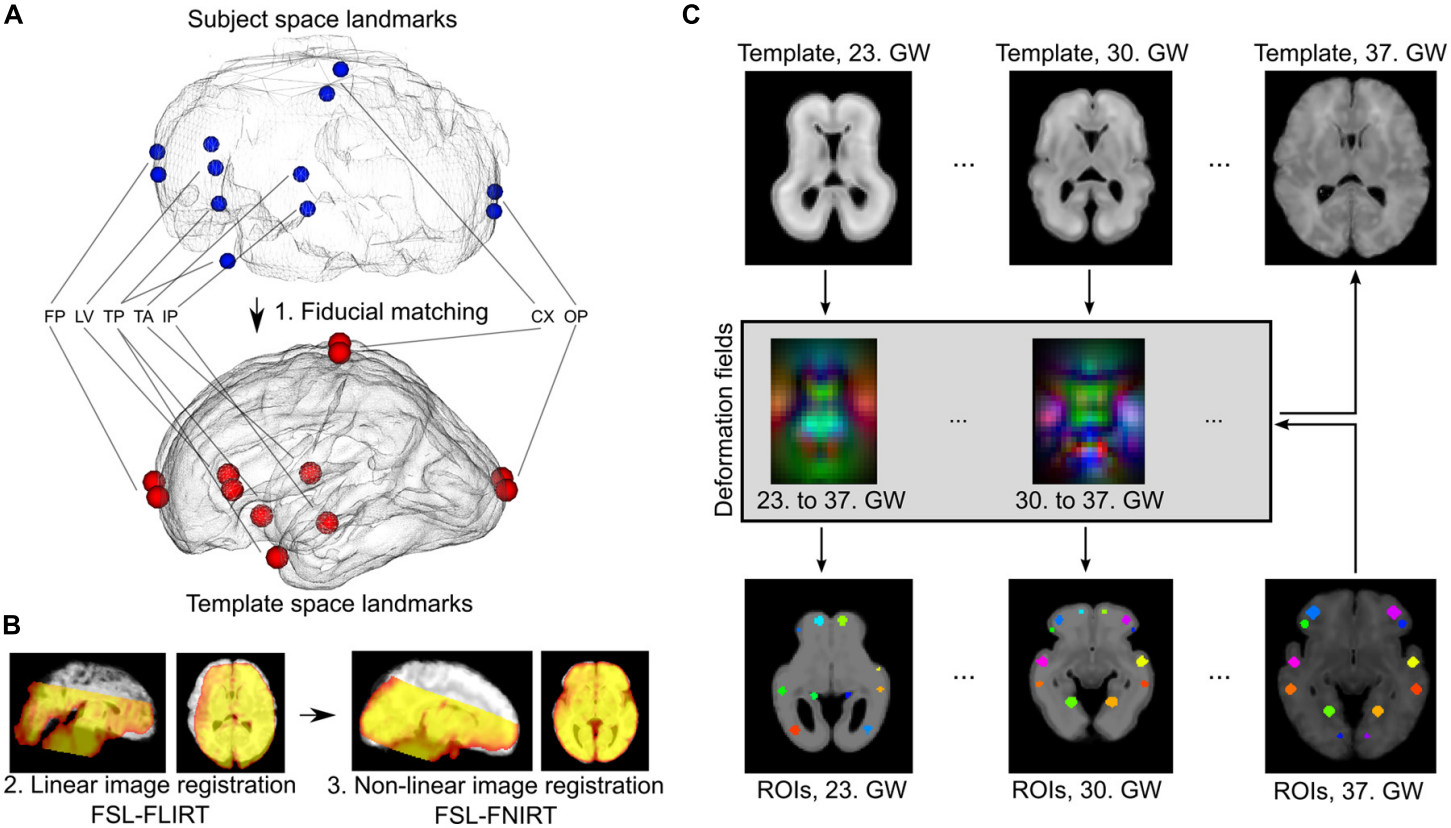
**Registration of fetal fMRI data to anatomical standard images and ROI propagation across developmental phases. (A)** Landmark-based initial registration was used to match the averaged BOLD fMRI images of fetuses and GW specific fetal template brains. FP, fontal pole; LV, lateral ventricles, frontal horn; TP, temporal pole; TA, thalamic adhesion (midline); IP, midline point of the interpeduncular cleft in the midbrain; CX, topmost point of the convexities; OP, occipital pole. **(B)** Second step of the fetal to standard image registration, carried out with intensity-based algorithms: linear (FLIRT) and non-linear (FNIRT) registrations in the FSL software. **(C)** Establishing GW-specific regions with a longitudinal label propagation approach. First row: for each GW from the 23rd to the 37th, a high-resolution anatomical template was used, between which pair-wise non-linear transformations were calculated (second row). These transformations were used to transform the ROI system into any GW (third and fourth rows).

Temporally averaged images of the fMRI data guided the registrations to achieve overlap with the T2-weighted templates. Each fMRI image was anatomically aligned to the respective GW-specific template image using the following procedure. We ensured proper orientation and coarse initial alignment by a landmark-based registration procedure using 12 manually annotated corresponding anatomical locations in the source and target images (**Figure 2**). We then relied on a two-step, intensity-based image registration in the FSL software (version 5.0, Linux 64-bit platform; Jenkinson et al., 2012): a linear, affine registration (FLIRT tool) followed by the non-linear matching of images (FNIRT algorithm), thereby coping effectively with the inter-individual anatomical differences. All further image processing steps, such as motion correction, noise reduction, band-pass filtering, and time-course extraction, were performed in the native space of the fMRI images, and the transformations were only used to propagate anatomically defined regions of interest into the subject’s space or propagate results through up-sampling to a reference space to aid visualization. Therefore, the possible confounding factor of image interpolation only affected the ROI system, and not the actual fMRI data.

### REGION OF INTEREST DEFINITION, FINDING CORRESPONDENCES ACROSS DEVELOPMENTAL STAGES

A ROI definition is critical when analyzing whole-brain structural or functional connectivity by node-based approaches, as it has a profound influence on network topology (Kelly et al., 2012). Conventionally, it is possible to obtain data-driven parcellations of a given territory or use anatomical priors to define gross landmarks. In the current study, we chose to use the latter. The 37th GW’s high-resolution template image was visualized to facilitate the localization of crucial areas that are commonly depicted in adult neuroimaging atlases, such as in the Harvard-Oxford Cortical atlas (Desikan et al., 2006). The labels either matched large surface structures (precentral, postcentral gyrus), or subdivisions of areas with known and assumed diverging connectivity profiles (anterior, lateral, posterior thalamus), or structures that corresponded to functionally defined regions (e.g., the calcarine cortex of the visual system).

**FIGURE 3:**
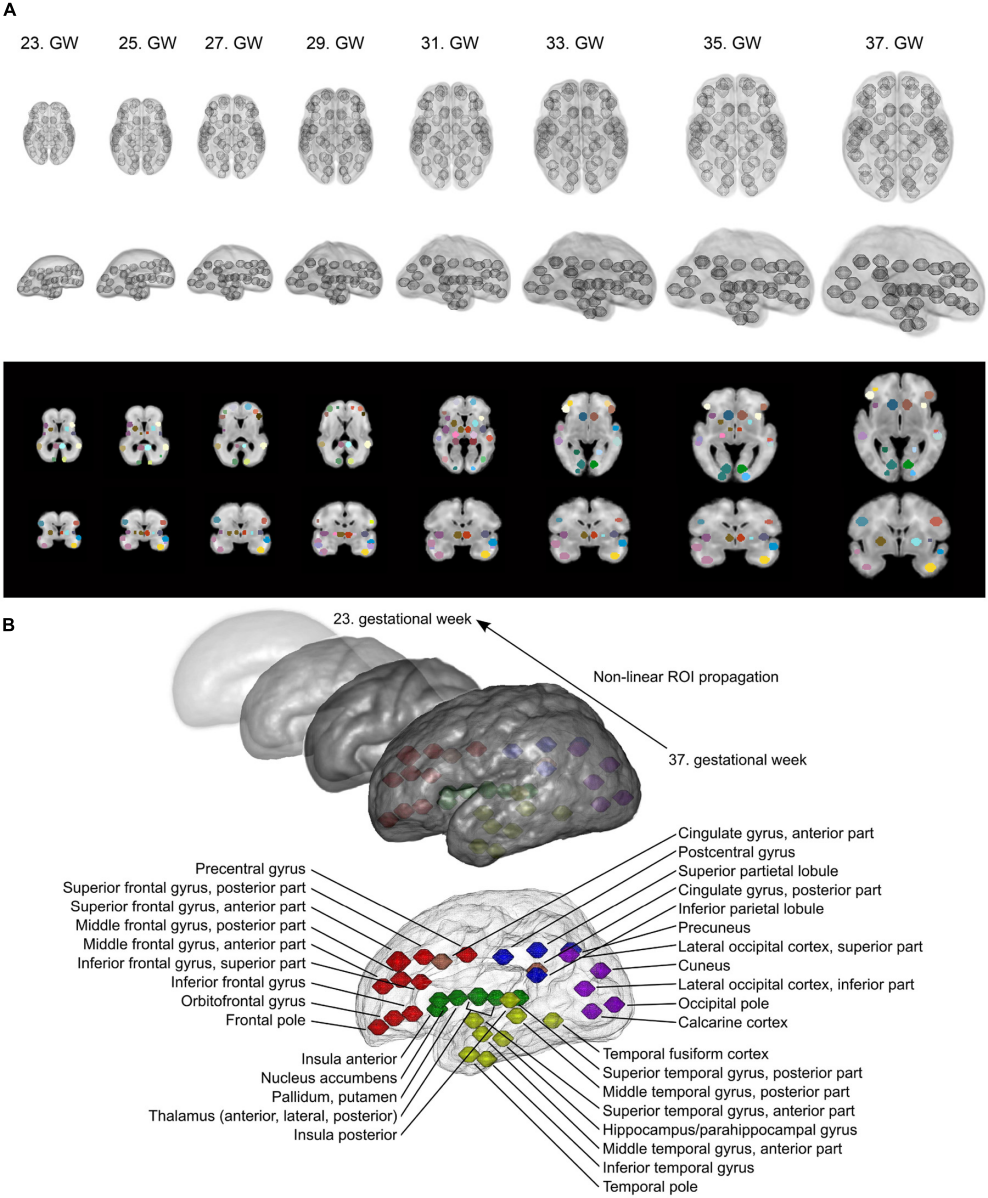
**Fetal brain atlas used for fMRI time-course calculation: ROI system and anatomical nomenclature. (A)** Illustration of the region propagation procedure. Using high-resolution anatomical templates, we marked 90 anatomically relevant regions on the fetal cortical surface, which was then propagated through the investigated gestational period. Three-dimensional renderings and cross-sectional anatomical images with ROIs are shown. **(B)** Anatomical nomenclature of the ROI system. Correspondence to major lobes or systems is marked with different colors: red = frontal lobe; blue = parietal lobe; purple = occipital lobe; yellow = temporal lobe; green = subcortical; pink = limbic/cingulate gyrus.

As the formation of cortico-cortical and cortico-subcortical circuits is not complete by the mid-fetal period, we placed anatomical regions onto the border of the subplate and the hypothesized future cortical plate position to capture presumably valuable signal from this region as well. In the reference ROI system, the ROI shape was spherical, mainly because little information is available about the volumetric extent of fetal cortical domains in the reference space. In 34.4% of the cases, the field of MR acquisitions did not cover the entire supratentorial area and the fetal cortex, and had limited cranio-caudal brain coverage. Examples for the ROI system, the ROI system matched to individual fetuses and the anatomical reference nomenclature is described in detail in **Figure 3**.

Our assumption was that the ROI system can be propagated into a developmental ensemble of brain reference images. Thus, ROI definition is only performed for the image having the best overall quality (i.e., in the 37th GW reference images, the secondary and tertiary sulci can guide the observer). Pair-wise registrations between the high-resolution templates were created in order to generate a collection of non-linear deformation fields that would feasibly match each GW’s space with the 37th GW’s template image. The non-linear registration was performed using “FNIRT” algorithm in FSL, with the registration parameters optimized to tackle the small scale of fetal brains. We illustrated this procedure in **Figure 2**. The correspondence between anatomical locations after the deformation in each GW template was checked by an observer with experience in neuroanatomy (András Jakab). The robustness of the ROI propagation method was visually checked by comparing the locations of ROIs with identifiable sulcal structures, such as the lateral, central, or parieto-occipital sulcus.

### CORRECTING THE CONFOUNDING EFFECTS OF FETAL HEAD MOTION AND NON-NEURONAL MRI SIGNAL

Significant head rotations and translations can occur during fMRI experiments due to the frequent, spontaneous fetal movements and maternal breathing. Excessive motion in terms of amplitude and duration served as exclusion criteria for fetuses. This decision relied on the subjective observer-driven selection in addition to other imaging artifacts, similar to the approach in previous works (Schöpf et al., 2012). After removing subjects, minor head motion was detectable in almost every case and displacements were estimated and corrected with the MCFLIRT algorithm in FSL (Oakes et al., 2005). This processing step comprised the rigid-body registration of images to the mid-scan frame and removal of those frames that showed excessive motion estimates (“scrubbing” frames with root mean square displacement larger than 2 mm; Power et al., 2012). As displacement induces spurious effects in signal intensity temporal characteristics, we used the motion parameters (translation and rotation) as first-level explanatory variables in signal filtering, as recommended in the literature (Weissenbacher et al., 2009).

**FIGURE 4:**
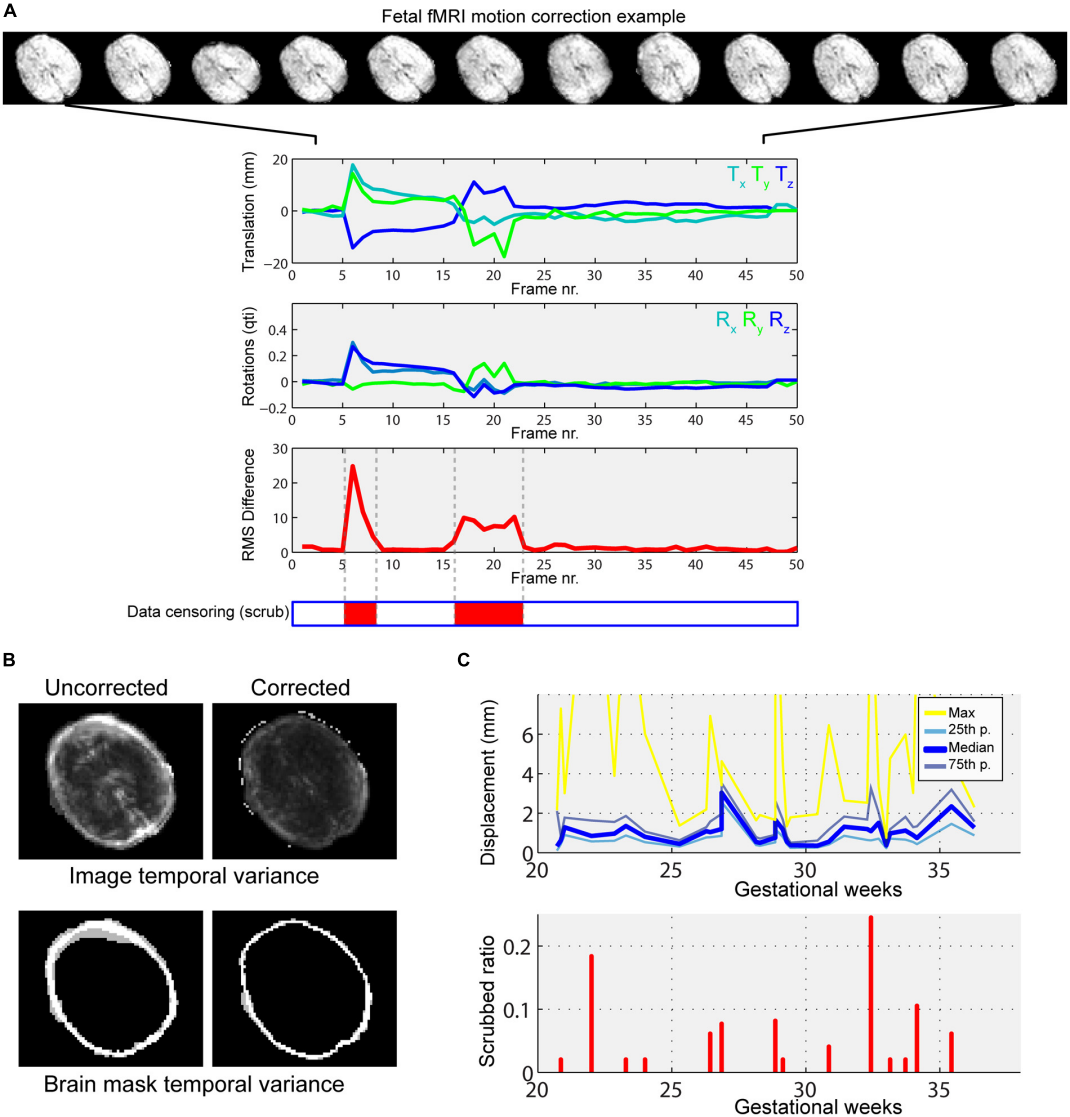
**Illustration of typical fetal in-scanner head motion patterns, correlation with gestational age. (A)** Fetal head motion during MR scanning is estimated by frame-to-frame linear registrations. We illustrate a fetus with excessive head movements, as indicated by the large peaks in the translation and rotational components. Total displacement was used to censor periods of the scan with large motion (middle panel, red markings). **(B)** The motion correction procedure reduces the temporal variance of peripheral brain voxels, as shown in the cross-sectional panels. Such regions of high, spurious temporal variance give rise to nuisance signals in the functional connectivity patterns. **(C)** Depiction of gestational age-dependent changes of head motion. Top: median, 25th percentile, 75th percentile and maximum displacements of the fetal head vs. GWs. Bottom: the ratio of censored data vs. GWs. In our study cohort, fetal in-scanner head motion does not show correlation with gestational age.

Furthermore, the variability of motion patterns can be influenced by gestational age. Hence we included per-subject motion as a covariate in higher-level analysis. This explanatory variable in the general linear model (GLM) putatively diminishes the effect of variable fetal motion among subjects at different developmental stages. We provide an example for typical fetal motion patterns, the effect of motion correction and the relationship to head motion to gestational age in **Figure 4**.

**FIGURE 5:**
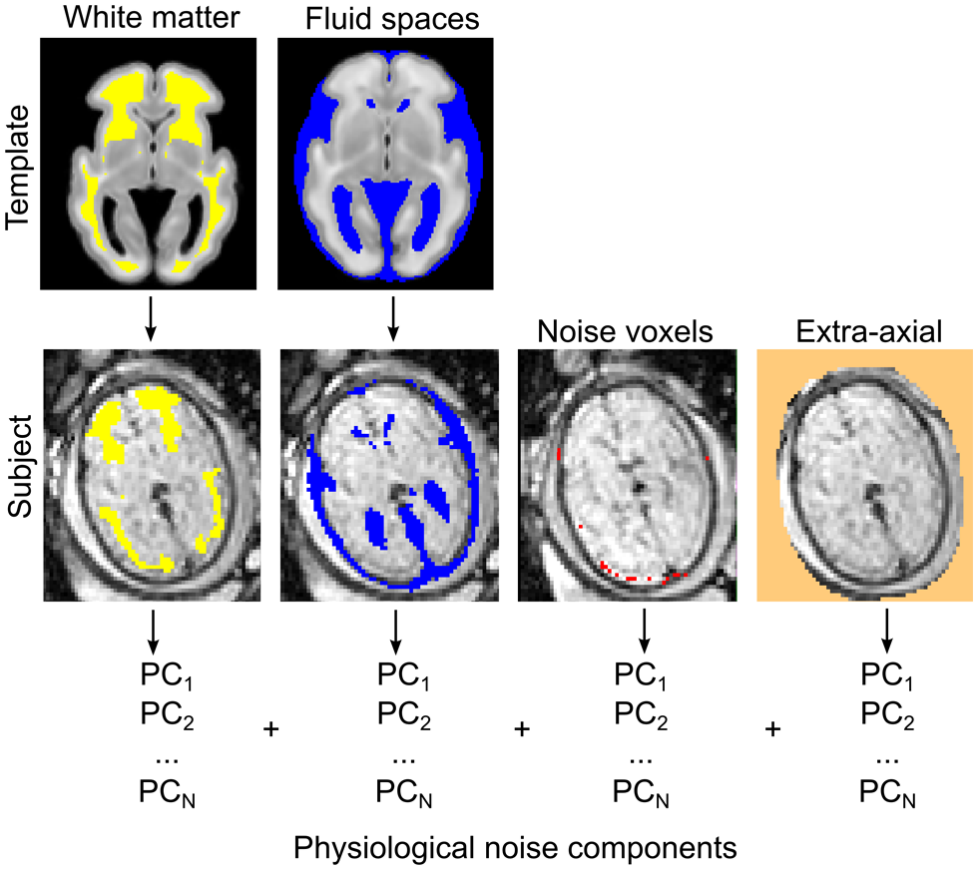
**Estimating physiological noise components for the correction of fetal fMRI signal using an adaptation of the CompCor approach. Top row:** white matter and CSF components are segmented from the GW-specific anatomical templates. Segmentations are transformed to individual subjects **(bottom row)** while the voxels with highest temporal variation, and other, extra-axial structures, are calculated from the data. These voxels are used to perform the component-based filtering of the nuisance signal, according to the CompCor method described by Behzadi et al. (2007). PCs, principal components.

The observed signal during fMRI experiments is known to be coupled with the underlying neural activity due to the BOLD phenomenon (Logothetis and Wandell, 2004). In contrast to the intuitive notion of *structural or axonal connection* in neuroimaging studies, a *functional connection* or co-activation between remote brain areas is assumed when the MR signal fluctuations in the low frequency range are correlated in the temporal domain (Lowe et al., 1998; Greicius et al., 2003). Functional connectivity is thus a purely statistical approach inherently prone to confounding factors, i.e., rhythmic variations in the MR signal not explained by the large-scale activation of neurons. The majority of studies employ a model in which confounding factors are attributed to image noise caused by circulation (Shmueli et al., 2007), respiration (Birn et al., 2008), head motion (Power et al., 2012), or MR equipment-related noise. During nuisance signal reduction, the neural signal is the residual after first-level regression analyses in which we use the time-courses of known non-neural origin and other nuisance variables. Here we adapted the CompCor approach to reduce noise (Behzadi et al., 2007). According to the CompCor noise reduction model, principal MRI signal components from the white matter, CSF, or other “noise” image voxels are used. Time-courses of nuisance signals were derived using anatomical priors, while voxels having the largest SD of signal intensity were segmented as noise voxels. The first five principal components (PCs) of these signals were entered into the confound regression procedure. We illustrate the nuisance confound regression procedure in **Figure 5**. A detailed description of the utilized fetal noise signal model is provided in the Supplementary Material.

### PORTRAYING THE DEVELOPING FETAL BRAIN CONNECTOME AS A COMPLEX GRAPH

We built undirected, weighted graphs representing intrinsic functional connectivity, with the set of longitudinally propagated brain regions as nodes. Connectivity value for a network edge was defined using the Z-transformed Pearson product-moment correlation coefficient between brain regions. No thresholding or binning of the network was performed. We used the adaptation of graph theoretical measures to weighted nets, as implemented by the Brain Connectivity Toolbox for Matlab (BCT, version: 2012. 12. 04) and described in (Rubinov and Sporns, 2010).

### TESTING HYPOTHESES ON COMPLEX NETWORKS: APPLICATION TO FETAL NEURODEVELOPMENT

To test our hypothesis about the developing brain, we modeled the temporal changes of brain connections as the effect of gestational time (gestational days) on the variance of individual connections, S_i,j_. Prior to this model, all connections were evaluated against simulated random brain graphs where the intrinsic functional connectivity were assumed to be explained by the spatial closeness of network edges and the consequent sharing of possible nuisance sources. This procedure is detailed in the Supplementary Material.

The application of univariate GLMs to each putative neuronal link (i.e., graph edge) raises the problem of mass multiple comparisons: in general, we cannot simply assume that such links are independent observations. Furthermore, correcting for the family-wise error rate (FWER) with the false discovery rate procedure would inherently result in a loss of statistical power due to the large number of univariate measurements, which, in our case, would mean 2415 individual connections (n_i,j_= 70, therefore the number of total connections is $\frac{70^{2}-70}{2}$).

The network-based statistics (NBS) method was utilized to tackle the multiple comparisons problem, as described by Zalesky et al. (2010) and implemented in the NBS Toolbox for Matlab R2012. This approach has the advantage of exploiting the internal connectional structure of brain graphs during the correction step for multiple comparisons, and potentially offers a substantial gain in statistical power. For the GLMs in our study, we provide the test statistics (*F*-test) for each connection in the graph and also for nodes where the test results were averaged over each edge connected with the node.

### DETERMINING LOCAL AND GLOBAL GROWTH CHARACTERISTICS

We aimed to determine the trend of overall functional network development (statistical threshold: *F*> 5, NBS-adjusted value) as well as for regional sub-networks. Thus, regional growth characteristics including, but not restricted to the connections in the frontal, parietal, temporal, and occipital lobes, short- and long-range connections, and thalamus connections were evaluated separately. The sub-network of a brain lobe was defined as all the connections that involve regions from that lobe.

Short- and long-range connections were defined by taking the Euclidean distance measures from the ROI-to-ROI distance matrix. For sampling short-range connections, the lowest 25th percentile of the distance matrix was used, while, for long-range connections, we used the highest 75 percentile. This categorization of short- versus long-range connections remained fixed during the gestation, and it was based on the Euclidean distances measured at the 37th week of gestation. We further evaluated the distribution of connectivity lengths across the gestation period.

We fitted two models on the observed network strength values over time: linear (first degree polynomial), and non-linear, sigmoid functions:

$$f{\left(x\right)}_{sub}=\frac{1}{1+e^{-\frac{x+a}{b}}}c+d$$

where f(x)_sub_ was the predicted value for the mean connectivity strength of the sub-network using the sigma model, x was the predictor variable (gestational age), and a, b, c, and d were the estimated coefficients. Using least squares fitting with robust estimation of outliers in the Curve Fitting Toolbox in Matlab R2012, we determined the goodness of fit for each selected sub-network and the global network. The least absolute residuals (LARs) approach was employed to estimate the statistical outliers during the regression procedure. The two models, i.e., linear and non-linear, were compared by their adjusted R-squared and SSE values. For sigmoid functions, it is possible to determine an inflation point, which is the x value (or the gestational age) that corresponds to the maximum point of its first derivative. Growth characteristics were further evaluated for global brain volumetric changes, head motion, signal intensity, and signal temporal variation.

A bootstrap sampling approach was utilized to test the statistical robustness of curve-fitting for each analyzed brain region. During bootstrapping, 50% of the observations were randomly selected, and the fitting of the linear and sigmoid functions was repeated over 1000 iterations. Two-sample *t*-tests were performed to compare the goodness of fit between linear and sigmoid fitting.

## RESULTS

### FETAL FUNCTIONAL MRI: DATA QUALITY AND AGE-RELATED CHANGES

**FIGURE 6:**
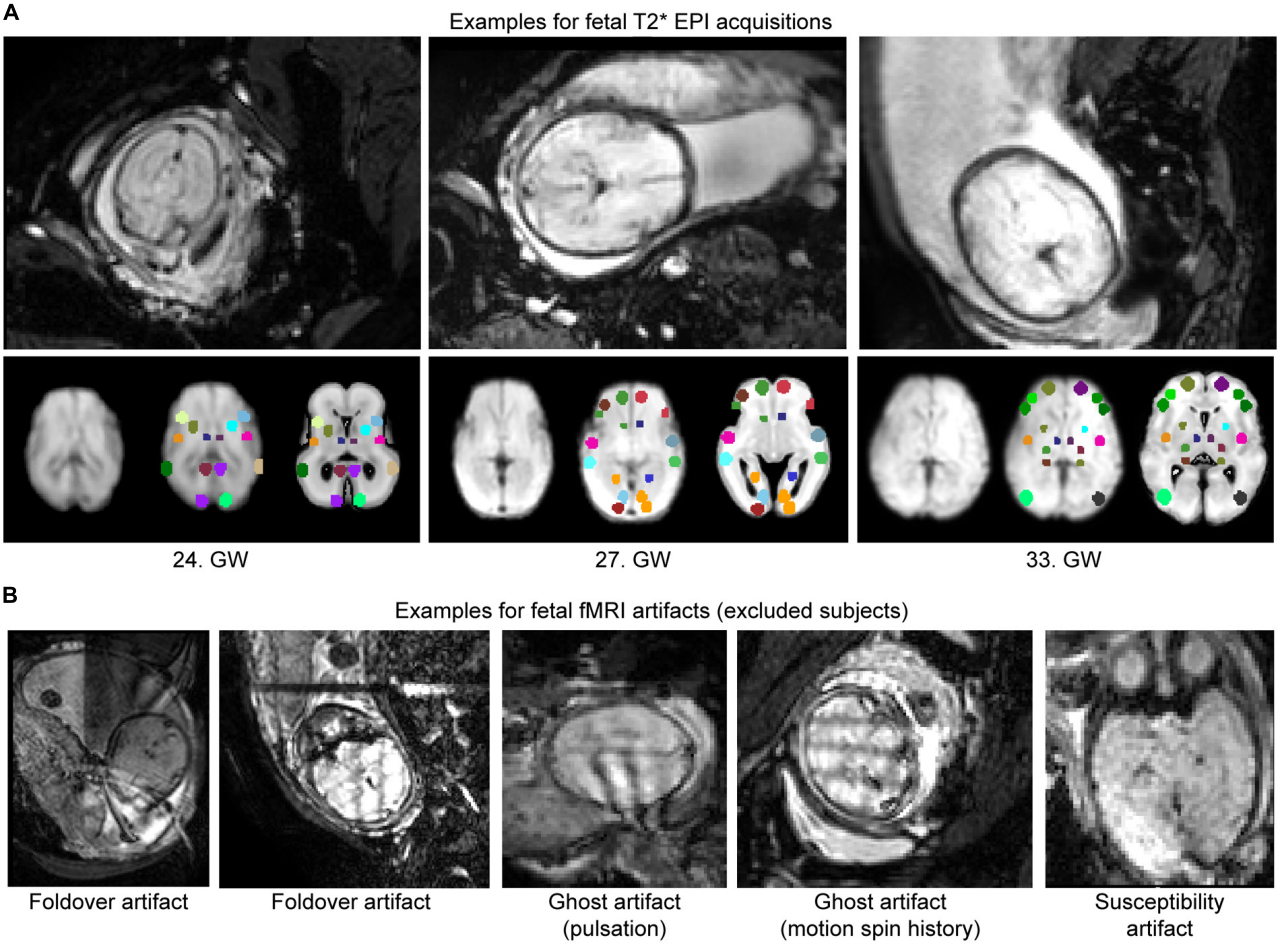
**Examples for fetal fMRI and the most common imaging artifacts. (A)** Fetal fMRI with full FOV. Bottom panels: standardized functional image, ROI system warped to the standardized image and the fetal atlas systems illustrated. We provide 1–1 examples from the GWs 24, 27, and 33. **(B)** Fetal fMRI is a challenging imaging modality with many artifacts to expect. We illustrate this by excluded subjects’ cross-sectional images. Examples for fold-over artifact, ghost artifact and susceptibility artifacts are shown.

Fetal fMRI is a challenging imaging modality, hindered by potential imaging artifacts. We give examples for fetal fMRI from our study database in **Figure 6**. After removing fMRI time frames with excessive fetal head motion, 46.4 ± 7.8 frames per subject were retained for analysis, while zero to nine frames were removed per case. The ratio of removed (censored) fMRI frames is illustrated in **Figure 4**. Motion correction reduced the image variance in the peripheral brain regions, which is commonly associated by movement artifacts (an example is provided in **Figure 4B**.). Head movement during the examinations (root mean squared displacement of brain voxels by translations and rotations) was not correlated with gestational age [mm, average: 0.643 ± 0.459 (range: 0.158–2.01); linear fit, *R*^2^ = 0.139, *p* = 0.448; Illustration: **Figure 4C**].

Next, we describe the potential age-dependent characteristics in our measurement, which may serve as confounding factors. The following quantitative parameters of fetuses changed over the course of gestation. Segmented fetal brain volumes showed significant linear growth [cm^3^, average: 266.7 ± 108.5 (range: 77–469.1); linear fit, *R*^2^ = 0.86, *p* < 0.001], and this correlation was similar to the increase in the total size of brain ROI over time [cm^3^, average: 6.954 ± 2.859 (range: 2.2–11.3); linear fit, *R*^2^ = 0.856, *p* < 0.001]. BOLD signal intensities decreased from the 21st to the 37th GW [signal intensity, average: 236.6 ± 66.4 (range: 127.7–387.9); linear fit, *R*^2^ = -0.207, *p* = 0.256]. This characteristic change in BOLD signal intensity can be attributed to the slight decrease in water content in fetal brain tissues during gestation and the consequent change of spin relaxivity (Chung et al., 2000). The temporal variation of the filtered and nuisance-corrected BOLD signal decreased over time [signal intensity variation, 6.378 ± 4.532 (1.02–19.06); linear fit, *R*^2^ = -0.3057, *p* = 0.088], although this decrease was not significant.

### EMERGING INTRINSIC FUNCTIONAL NETWORKS

Network-based statistical inference analysis was performed for 2415 different functional connections (i.e., upper triangle of the brain connectivity matrix; Zalesky et al., 2010). Permutation testing revealed that 850 were different from a random graph, and the intrinsic functional connectivity values of 114 region pairs significantly correlated with gestational age after correcting for multiple comparisons. A linear and a non-linear growth model were tested for the relationship between gestational age and functional connectivity. The variation of each statistical parameter was quantified in terms of the uncertainty calculated during a bootstrapping sampling procedure.

**FIGURE 7:**
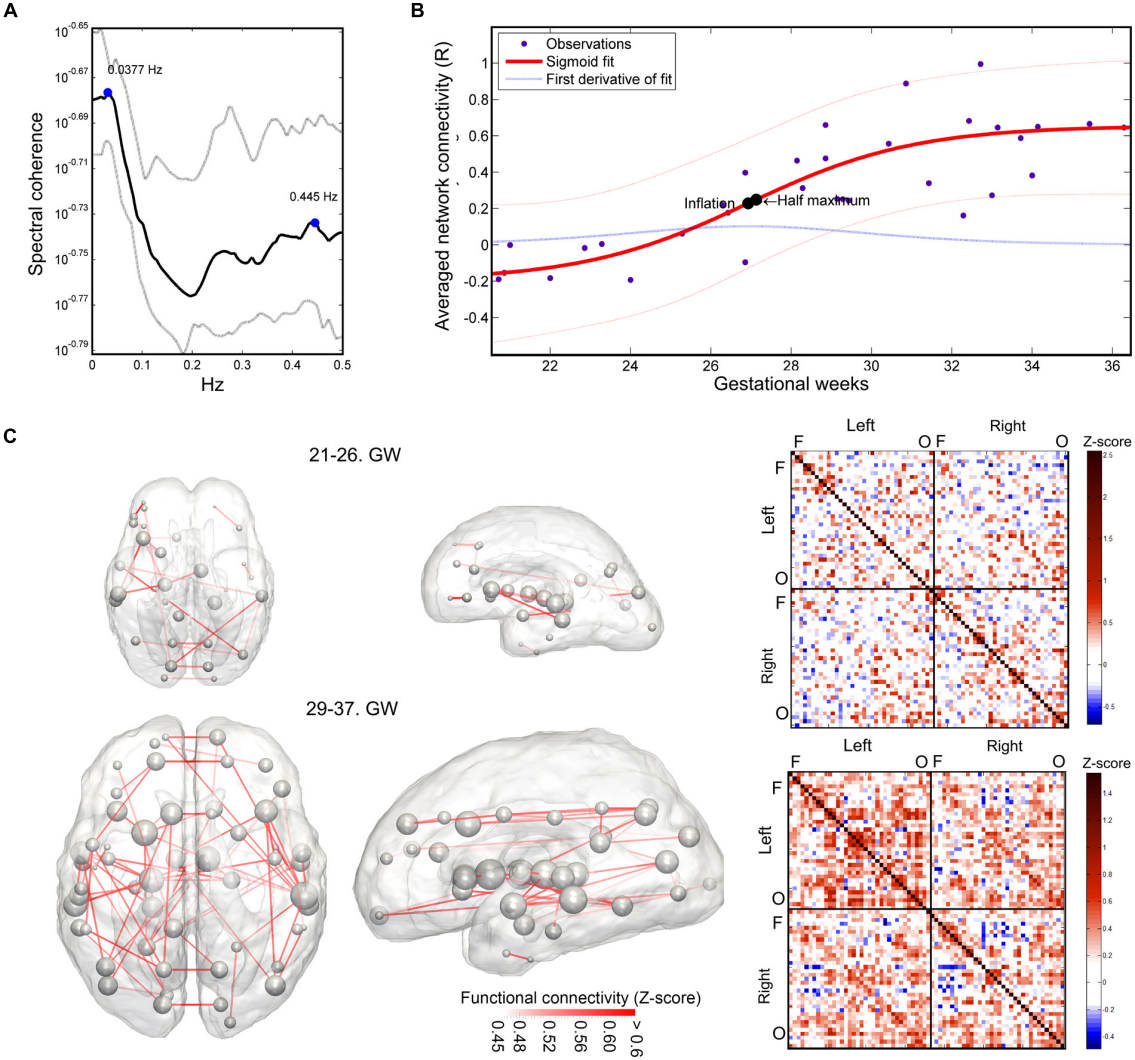
**Results of the functional connectivity analysis: spectral coherence of time-courses, developmental trajectory of developing fetal intrinsic functional connectivity. (A)** Spectral coherence analysis of fetal fMRI time-courses. The mean coherence plot (black line) was derived for all pairs of fetal functional time-courses at all GWs. Gray line depicts the interquartile range of observation across subjects. The plot is dominated by two peaks, representing low-frequency synchronicity at 0.0377 Hz and possible noise correlation at 0.445 Hz. Bold line: mean coherence, dotted lines: SD. **(B)** Development of the fetal functional brain connectome, gestational age-dependent distribution of functional connections. We illustrate the sigmoid curve-fitting on individual average network connectivity strength measurements. Each point in the graph refers to the mean intrinsic functional connectivity of a fetus, which calculation was restricted to the values that were found to be significantly associated with gestational age. Half-inflation point of the curve is found at the 27th GW and it depicts the gestational age in which the maximum increase of intrinsic functional connectivity is found. **(C)** Developmental expansion of the fetal functional brain connectome, visualization as matrices and 3D graphs. Two stages of the development are depicted: prior to the presumed expansion of functional connectivities (21st–26th GWs) and after this period. This dichotomy was defined using the average intrinsic functional connectivity values **(B)**. The transparence of each edge in the 3D visualization (left images) refers to the population mean of the intrinsic functional connectivity (*Z*-score). The size of nodes in the network depiction is proportional to the sum of connectivity values over all edges that are connected to them. Group-mean network edges were visualized using a threshold of *Z* = 0.45. Graphs were overlaid on anatomical templates from the 23rd and 37th GWs. The mean connectivity networks are also visualized as connectivity matrices, Fs, frontal regions, Os, occipital regions.

The mean intrinsic functional connectivity of the entire brain network showed a significant increase over gestation (linear model, *R*^2^-adjusted: 0.623 ± 0.0467; SSE: 0.93 ± 0.16; sigmoid model, *R*^2^-adjusted: 0.66 ± 0.063; SSE: 0.875 ± 0.167), and the sigmoid function described the relationship between GW and connectivity more powerfully (difference of SSEs, *p* < 0.001). Until the 25th GW, the mean network connectivity remained close to zero (average connectivity strength range from the 21st–26th GW: -0.163–0.0525). The inflation point of the non-linear strengthening of functional connections was estimated at 26.59 ± 1.19 weeks. The period of connection development in which the connection strengthening rate is larger than 90% of the mean inflation rate was defined as the “expansion period.” This interval was calculated from the first derivative of the fitting function. The expansion period of the total functional connectivity network was observed between GWs 25.58–28.69. We illustrate the increase of functional connectivity in **Figure 7B**.

Using this concept of expansion, we provide visualizations of the mean fetal functional network structure for pre- and post-expansion periods, and calculated the connectivity values for each of these states of functional connectivity development in **Figure 7C**.

The average intrinsic functional connectivity values in the fetal brain graphs during the expansion period (GW 26–29) were significantly higher than before the 26th GW (pre-expansion: -0.0696 ± 0.0834; expansion: 0.234 ± 0.148; *p* = 0.000173). This difference was also significant for values before and after the expansion state (pre-expansion: -0.0696 ± 0.0834; post-expansion: 0.385 ± 0.159; *p* = 0.036). Our results are summarized in **Table 1**. Between the 21st and 26th GWs, the mean functional network architecture is dominated by occipital and temporal connections, while frontal, parietal, or interhemispheric connections are sparser. After the expansion period (29–37th GW), we observed the increase of functional connectivity strength in the parietal and frontal lobes and long-range correlations between the frontal and temporal lobes.

**Table 1:** Prenatal development of intrinsic functional connectivity and changes in the region-to-region Euclidean distances.

	Average network connectivity, region-to-region distances (mm)	Average functional connectivity strength of network
Pre-expansion (GW 21–26; *n* = 8)	24.726 ± 1.922 (21.232–27.186)	-0.0696 ± 0.0834 (-0.163–0.0525)
Expansion (GW 26–29; *n* = 8)	27.28 ± 1.608 (25.344–30.526)	0.234 ± 0.148 (-0.0669–0.38)
Post-expansion (GW 29–37; *n* = 16)	26.612 ± 1.459 (23.106–28.626)	0.385 ± 0.159 (0.147–0.6094)
Difference: pre-expansion vs. expansion	Increase: 2.554; *p* = 0.0121	Increase: 0.304; *p* = 1.7296e-4
Difference: post-expansion vs. expansion	Increase: 1.885; *p* = 0.0135	Increase: 0.151; *p* = 0.036

*Functional connectivity refers to the Pearson product moment correlation coefficient of the signal time-courses of two brain regions. The average functional connectivity strength is the average correlation coefficient of the network edges that were found to be significantly correlated with gestational age. For optimal comparison, the reported Euclidean distances were fixed for each network edge and these values reflect the distances found at the 37th week of gestation.*

### HETEROGENEOUS AND REGION-SPECIFIC DEVELOPMENTAL CHARACTERISTICS

After separating the entire connectivity network into regions according to brain lobes, we evaluated the developmental trajectories of within-lobe, interhemispheric, and thalamo-cortical connections. Here we use the term “connectivity strength” for the average intrinsic functional connectivity values of a given group of network edges. The estimation of expansion periods revealed a sequential order for how regional functional connectivity becomes increasingly strong. The non-linear sigmoid expansion better described the regional increase of intrinsic network connectivity strength (**Table 2**). The correlation between the gestational time and the connectivity observations was high, with adjusted *R*-squared values ranging from 0.45 (thalamus connections) to 0.64 (interhemispheric connections).

**Table 2:** Regional characteristics of prenatal intrinsic functional connectivity development: results of the regression analysis.

	Linear model			Sigmoid model				Linear vs. sigmoid
	*R*^2^	Adjusted *R*^2^	SSE	*R*^2^	Adjusted *R*^2^	SSE	Inflation time	
Overall network	0.639 ± 0.045	0.624 ± 0.047	0.93 ± 0.161	0.66 ± 0.064	0.613 ± 0.073	0.878 ± 0.168	26.589 ± 1.196	Sigmoid, *p* < 0.0001
Occipital connections	0.513 ± 0.059	0.492 ± 0.061	1.378 ± 0.219	0.538 ± 0.071	0.473 ± 0.08	1.312 ± 0.251	24.826 ± 1.589	Sigmoid, *p* < 0.0001
Frontal connections	0.563 ± 0.062	0.544 ± 0.065	1.187 ± 0.194	0.649 ± 0.06	0.6 ± 0.069	0.954 ± 0.153	26.434 ± 0.477	Sigmoid, *p* < 0.0001
Temporal connections	0.505 ± 0.054	0.484 ± 0.056	1.601 ± 0.254	0.541 ± 0.063	0.477 ± 0.071	1.468 ± 0.269	25.993 ± 0.929	Sigmoid, *p* < 0.0001
Parietal connections	0.566 ± 0.081	0.547 ± 0.084	1.381 ± 0.31	0.602 ± 0.058	0.547 ± 0.066	1.297 ± 0.22	27.477 ± 0.786	Sigmoid, *p* < 0.0001
Thalamocortical and subcortical connections	0.504 ± 0.0548	0.483 ± 0.057	1.967 ± 0.317	0.519 ± 0.067	0.453 ± 0.077	1.919 ± 0.312	26.31 ± 1.75	Sigmoid, *p* < 0.0001
Short-range connectivity	0.509 ± 0.048	0.488 ± 0.05	1.642 ± 0.251	0.557 ± 0.065	0.495 ± 0.075	1.471 ± 0.251	27.69 ± 0.942	Sigmoid, *p* < 0.0001
Long-range connectivity	0.673 ± 0.052	0.657 ± 0.054	0.532 ± 0.104	0.545 ± 0.07	0.481 ± 0.08	1.471 ± 0.293	25.647 ± 1.445	Linear, *p* < 0.0001
Interhemispheric connections	0.663 ± 0.044	0.649 ± 0.046	0.904 ± 0.144	0.687 ± 0.046	0.643 ± 0.053	0.853 ± 0.132	26.416 ± 1.014	0.084

*Two models were tested, we evaluated whether connectivity development was better described with a linear or a non-linear sigmoid growth model. Variance during the bootstrapping analysis is given by the SD. Group comparisons were performed using two-sample, two-sided Student *t*-tests, assuming equal variances.*

The inflation time shows a heterogeneous pattern across the fetal cortex. The inflation time of the occipital connections was the earliest (*R*^2^-adjusted: 0.47 ± 0.08, inflation point: 24.83 ± 1.59 GW), while the inflation time for the parietal connections was the latest in the sequence (*R*^2^-adjusted: 0.55 ± 0.07, inflation point: 27.48 ± 0.79 GW). The occipital expansion of functional connectivity was followed by the temporal, frontal, and parietal lobes. Thalamus-cortex interconnections displayed trends of development similar to that of the overall network (expansion of overall network: 26.59 ± 1.19 GW; expansion of thalamic connections: 26.31 ± 1.75 GW).

### COMPARING SHORT- AND LONG-RANGE CONNECTIVITY

**FIGURE 8:**
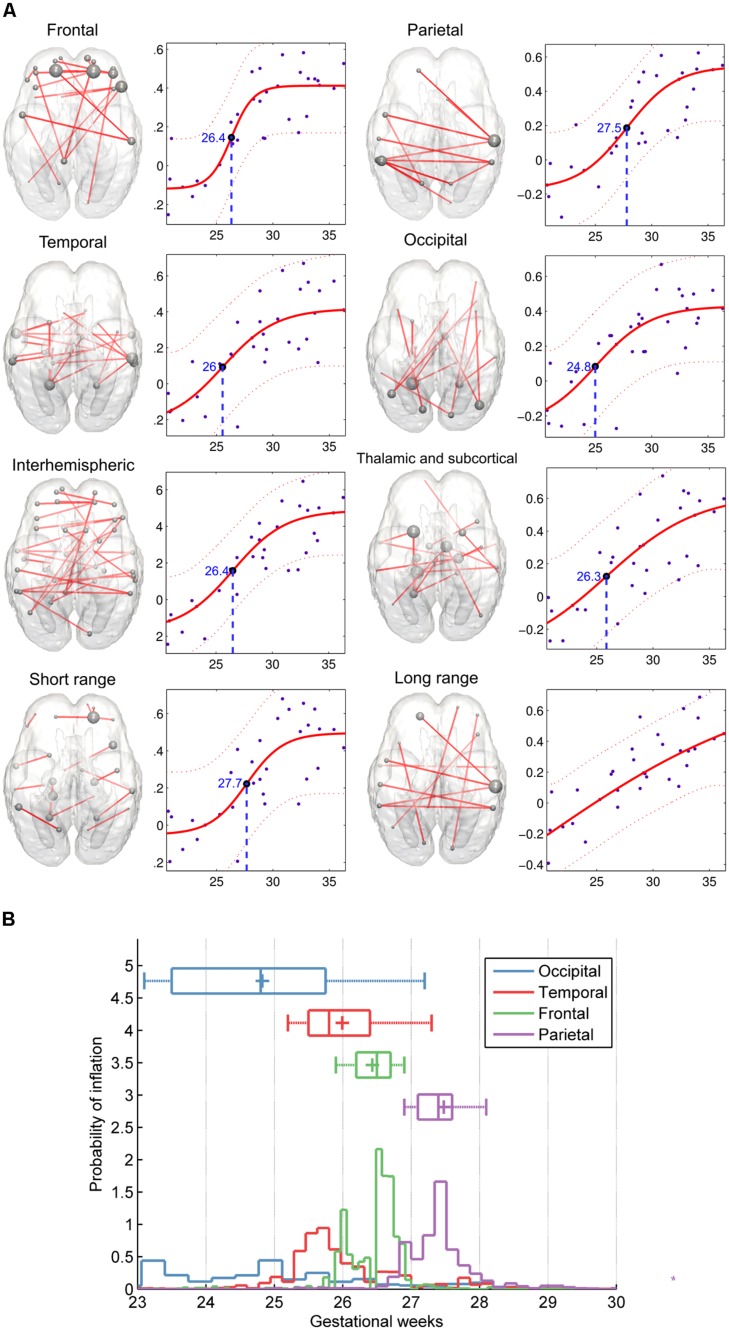
**Region-specific development of functional connectivity in the fetal brain. (A)** In these visualizations, a regional sub-network was defined to include all connections within a brain lobe, and these were restricted for the edges which connectivity values showed significant correlation with gestational age. Plots depict significant age effects on functional connectivity within each sub-network. Components of sub-networks are depicted as graphs, overlaid onto a 25th-GW brain template model. In this form of visualization, graph edges refer to a functional connection (*Z*-score) between two areas, while the brain areas are represented as nodes. Node size refers to the sum of its connections, weighted by the functional connectivity. **(B)** Regional timing of functional brain connectivity expansion during the late second and third trimester, variability of the observed inflation times during the bootstrapping procedure. According to the hypothesis that functional connectivities exhibit non-linear growth characteristics rather than a linear trend during the mid-fetal period, it is possible to determine the gestational age of inflation by sampling. 50% of the measurement points were sampled randomly during this procedure, and the relative probability estimates of the inflation points of the fitted functions are depicted against the gestational age. The distribution of sampled connections indicates a sequence of occipital, temporal, frontal, and parietal development of intrinsic functional connectivity values.

A further distinction was made according to the region-to-region physical (Euclidean) distances: short- and long-range connections were analyzed separately. Maximum strengthening of all short-range connections occurred at the 27.69 ± 0.94 GW, although we were unable to determine the same for long-range functional coherence where linear strengthening was discovered (SSE of linear model: 0.53 ± 0.1; SSE of non-linear model: 1.47 ± 0.3, *p* < 0.0001). Numeric data of the revealed order of development are summarized in **Table 2**. We provide a visualization of the core regional networks, the developmental curves and the estimated sequence of functional connectivity inflation in **Figure 8**.

After the 26th GW, the proportion of long-range connections becomes significantly higher, as reflected by the larger Euclidean region-to-region distances within the reference network (**Table 1**).

The sigmoid strengthening described the functional connectivity of short-range connections significantly better than the linear, but this observation did not hold for long-range connections. Instead, it appears that long-range connectivities gradually increase from the beginning of the observed gestational period.

### SPECTRAL COHERENCE ANALYSIS OF FETAL FUNCTIONAL MRI SIGNALS FROM THE CORTICAL PLATE

Magnitude-squared spectral coherence of BOLD recordings characterized low frequency synchrony of intrinsic signal fluctuations in the fetal brain. Prominent frequency peaks of signal synchronicity were found at 0.0377 ± 0.0599 Hz and 0.427 ± 0.068 Hz, and peak heights were 0.255 ± 0.096 and 0.219 ± 0.047, respectively. This pattern remained consistent at all observed gestational ages. Similar to adult fMRI measurements, it can be assumed that the lower frequency peak corresponds to the neural component, while the higher frequency component is likely to be explained by cardiac effects on the signal (calculated frequency, beats per minute: 144 ± 28, range: 120–216). We illustrated the average spectrum of time-courses and the spectral coherence in **Figure 7A**.

## DISCUSSION

### CHARACTERISTICS OF THE DEVELOPING FETAL FUNCTIONAL CONNECTOME

In this work, we have employed fMRI to portray the development of the fetal functional brain connectome. The rate of signal synchronicity increase is highest at the transition from the second to the third trimester, with the peak around the 26th GW. MRI signal fluctuations were first synchronized in the occipital lobe (e.g., primary visual cortex), followed by the temporal, frontal (including the motor cortex), and parietal lobes. When looking for the optimal characteristics to describe the increase of

intrinsic functional connectivity, observations followed a non-linearly increasing trend, the only exception to which were the long-range connections. The proportion of short- and long-range connections was not homogeneous in the evaluated course of gestation: after the expansion period (26th to 29th week), more long-range connections could be observed.

### PRENATAL DEVELOPMENT OF FUNCTIONAL BRAIN CONNECTIVITY FOLLOWS POSTERO-ANTERIOR TIMING

The heterogeneous timing of network development across the cortex relates to findings regarding the network variability across adult subjects. Mueller and co-authors found that parietal regions—in particular the association cortex—exhibit high variability compared to primary regions, such as the sensorimotor system (Mueller et al., 2013). The timing suggests that the regions with high inter-individual variability are those regions whose connectivity structure is developed relatively late during gestation. Similarly, these late-developing regions are phylogenetically young cortical areas that exhibit the strongest cortical expansion during late evolution, when comparing the macaque and human cortex (Hill et al., 2010). Buckner and Krienen (2013) discuss the timing of primary cortical areas and subsequent areas, such as the association cortex, relating the complex long-range networks with relatively weak organizational coherence across individuals to the rapid expansion of the corresponding cortical areas during evolution. Although we do not answer the question of whether this is mirrored during early individual brain development, the results suggest that the fetal brain appears to exhibit timing patterns similar to those of cortical evolution.

### EMERGENCE OF FETAL NEURAL ACTIVITY: BIOLOGICAL BACKGROUND

The second and third trimester of gestation is critical for the healthy development of cerebral pathways and for the normal functioning of the newborn brain. Here, we interpret findings in the context of major events of telencephalon development within this time frame. Cortical neurons begin to receive their first afferent inputs from the thalamus through a multiple-step process that involves the establishment and resolution of transient connections through the subplate (Price et al., 2006; Kostovic and Judas, 2010). The subplate constitutes a distinct layer of the fetal cortex (Kostovic and Molliver, 1974) and contains a heterogeneous set of early post-migratory neurons, which show the most advanced and mature electrophysiological characteristics compared to other neurons of the fetal brain (Moore et al., 2009). Experimental data indicate an essential role of GABAergic subplate neurons in the generation of early synchronized network activity, and thus, in the establishment of normal neuronal circuits and a regular columnar architecture of the developing cortical plate (Zhang and Poo, 2001; Lopez-Bendito and Molnar, 2003; Kanold, 2009; Luhmann et al., 2009; Judas et al., 2013). Electrical discharges in the human cortex can be demonstrated *in vitro* (Penn and Shatz, 1999; Moore et al., 2009). Moreover, correlates of spontaneous network activity can be identified by early human preterm electrophysiology.

The maturation of thalamocortical afferentation is accompanied by the evolution of a more complex electroencephalography pattern (Vanhatalo and Kaila, 2006), including delta brushes from the 28–30th GWs onward (Khazipov and Luhmann, 2006). Thus, early cortical maturation occurs mainly independent of sensory input, and is driven by spontaneous activity, amplified through subplate neurons (Luhmann et al., 2009). Due to the high water content in the extracellular matrix and the low number of neurons, the subplate displays distinct MRI features, as described *in vitro* and *in vivo* (Maas et al., 2004; Prayer et al., 2006). A spatial and temporal regional morphological variation during development has been described. In the dorsolateral cortical areas, receiving massive cortico-cortical and thalamocortical input, the subplate shows the thickest dimensions, whereas it is diminished in size in the mesocortical regions (Kostovic et al., 2002). There is a developmental peak between 27 and 30 GWs, followed by a stage of dissolution after 30 GWs. Thus, the morphologic dimensions of the fetal subplate mirror the dynamics and grade of the regional connectivity of somatosensory and association cortices. This spatiotemporal anatomic trajectory is in accordance with the results of our functional imaging-based global effects analysis (**Figure 4**). The rapid and significant increase in connectivity between 24 and 31 GWs, with a half-inflation point at 27 GWs correlates with the period of maximum growth of the human fetal subplate and increasing synaptogenesis in the cortical plate. We were able to describe a stage of stabilization of the connectome after 31 GWs, which parallels the process of subplate dissolution and the consolidation of existing networks. The general hierarchy of myelination starts with sensory pathway myelination before the motor and association pathways (Flechsig, 1920). Although myelination mainly occurs postnatally, we were able to demonstrate that functional connections develop first in the occipital and temporal areas at around the 25th GW, whereas the frontal lobe connectivity evolves later. This is consistent with recent neuroimaging and neuropathology data showing an earlier maturation of the optic and acoustic radiations, compared to the frontal lobe pathways traveling through the anterior limb of the internal capsule (Yakovlev and Lecours, 1967; Kinney et al., 1988; Dubois et al., 2008). Trivedi et al. (2009) used diffusion tensor magnetic resonance imaging to demonstrate the region-specific maturation of the fetal brain, concluding that diffusion anisotropy quantifies the microstructural orderedness that reflects the peaking activity of neuronal migration. Coherent with the functional developmental timing in our study, they revealed an initial occipital and temporal increase and peaking of anisotropy.

Moreover, we reported that long-range and interhemispheric connectivity shows a linear increase during development, as opposed to the rapid dynamics of short-range connectivity within certain brain lobes. As long-range association pathways as well as commissural pathways follow a steady and linear developmental trajectory, the dynamics of our functional imaging-based connectome seems to reflect the divergent properties of these processes.

### RELATIONSHIP TO PREVIOUS FINDINGS

*In utero* fMRI came to the forefront only in the last decade to assess stimulation-related activity (Gowland and Fulford, 2004; Jardri et al., 2008) or activation in resting conditions (Schöpf et al., 2012). To date, only a small number of studies have employed this method for the portrayal of functional ontogenesis in normal or pathological circumstances.

A possible way to extend our knowledge in this field is to adapt imaging techniques that are widely used in humans after birth. In this regard, *in vivo* neuroimaging techniques, such as diffusion tensor MR imaging (DTI, Johansen-Berg and Rushworth, 2009; Dennis et al., 2013; Yoshida et al., 2013) or BOLD fMRI have already contributed to significant findings in human developmental neuroscience. The analysis of fetal functional neuroimaging data as an emerging network can be viewed as an extension of previous single-subject or group-based analytical approaches (Schöpf et al., 2012; Thomason et al., 2013).

Observations of fetal behavior *in utero* (Joseph, 2000; Morokuma et al., 2013) outline a fine-tuned arrangement of how functioning neural ensembles emerge that serve basic capacities, like somatomotor functioning, auditory responses, plasticity, or regulatory mechanisms (D’Elia et al., 2001; Partanen et al., 2013a). Evidence for the emergence of fetal auditory processing and simple learning capacities mostly comes from electrophysiological measurements (Holst et al., 2005; Partanen et al., 2013b) or task-related functional imaging (Jardri et al., 2012).

Similarly, fMRI of the immature neonatal brain is restrictive for the characterization of *typical* development: observations are mostly limited to the 26–40th weeks of gestation when the prerequisites for sensory-driven processing are assumed to be already in place (Doria et al., 2010).

Our findings of increasing interhemispheric intrinsic functional connectivity are in line with previously performed studies utilizing prenatal MR imaging, with a notable difference that previous works reported qualitative findings on the emerging activity, or used linear growth models for selected connections (Schöpf et al., 2012; Thomason et al., 2013). The sequence of functional connectivity increase was recently reported to follow a medial–lateral and posterior–anterior trajectory, which is partially supported by our observation of an occipital-temporal-frontal course of network emergence. Using ICA to map the temporo-spatial components of prenatal brain activity, Schöpf et al. (2012) revealed consistent bilateral occipital networks as early as the 22nd GW. Further reproducible components were observed bilaterally in the frontal lobe and unilaterally in the temporal lobe, and, notably, parietal networks were absent. In our developmental model, the pre-expansion period before the 26th week was characterized by bilateral occipital, unilateral temporal connections, and the presence of inter-hemispheric long-range synchrony between non-homotopic areas. The low-frequency MRI signal fluctuations were found to be the most coherent in the 0.01–0.1 Hz range, which matches the commonly assigned filter intervals in adult studies and other prenatal fMRI analyses. In this work, we relied on a more intricate representation of the developing brain activity, compared to previous works on prenatal data: a graph model and network-based inference approach was chosen to capture the overall network properties in a statistically sound framework. In addition, a careful preprocessing pipeline was used to lessen physiological noise and confound the effects of fetal head motion (Power et al., 2012).

The analysis of the developing functional connectome and macroscopic-scale network architecture was previously elucidated using imaging data from immature neonates. According to a report by Doria et al. (2010) preterm neonates seem to exhibit distributed functional connectivity networks at birth. A longitudinal study by Smyser et al. (2010) discovered a variety of network components that resembled their adult counterparts as early as the 26th GW. A consistent component in similar studies is the *default mode network*, whose prenatal existence has been questioned (Lee et al., 2013). This interrogation of developing resting-state functional networks suggests that the period of the most rapid synaptogenesis triggers spontaneous brain activity that is organized into networks, and such networks are similar in spatial extent to those observed in newborns.

By measuring the signal intensity changes coupled with blood oxygenation levels, fMRI indirectly characterizes the time-course of slowly fluctuating neural activation (Logothetis and Wandell, 2004). Physiological measurements suggest that fMRI activations are neuronally driven and more strongly coupled to dendritic activity than action potentials (Lauritzen, 2005). The known mid-gestational shifts of electrical activity in the human cortex correlate with the reported periods of functional connectivity expansion, although a definitive conclusion cannot yet be drawn due to the limitations of *in vivo* imaging technology. Spontaneous brain activity in preterm neonates remains questionable as a model for normal, *in utero* development (Damaraju et al., 2010); a more faithful description about the electrophysiological maturation in healthy subjects would be optimal to explicate our findings on functional activity. So far, such an investigation on normally developing human subjects is not feasible and this problem remains unsolved.

### STUDY LIMITATIONS

A number of factors limit the experimental support of our work to describe emerging brain function in human fetuses. Here we report three major categories of possible limitations in fetal fMRI studies: (1) origin of fMRI signals in the developing brain, (2) fetal head motion and age-related confounds, and (3) finding optimal anatomical correspondences during gestation and ROI system creation.

Colonnese and Khazipov (2012) advised a more careful interpretation of pre- and perinatal fMRI studies on the grounds that the coupling of the BOLD signal and neuronal activity is immature or unknown. The relationship between the MRI signal and the underlying neural activity is the topic of extensive debate. However, there is converging evidence that functional connectivity can be explained or predicted by mono- and polysynaptic cortico-cortical interconnections (Honey et al., 2009). Furthermore, spontaneous low-frequency activations in the fetal sensory areas are possibly driven by mechanisms different than that in the adult brain, serving the development of the cortex and not endogenous processing.

Fetal in-scanner head motion is a challenging issue in fMRI, and here in our study this was the primary criteria for excluding otherwise normally developing fetuses. For fMRI experiments with long TR, fetal motion can occur between slices as well, requiring special algorithms for motion correction. In our study, we did not reveal significant dependency of fetal motion patterns with gestational age, although in a larger, unfiltered cohort, this can be expected. Therefore we suggest including total subject-level head displacement as a higher level explanatory variable in group comparisons. As head motion causes not only voxel displacement but signal intensity changes as well, it is important to include motion confound variables in the nuisance regression step.

Here we summarize the following age-related confounds that can hinder the interpretation of our current results. First, fetal brain size significantly grows during gestation, therefore any trend in functional connectivity increase can be a result of merely observing larger ROI. Another consequence of the brain growth is that ROIs are naturally closer to each other in a young brain and this may result in a bias toward stronger short-range connections. In our study, fMRI signal variance did not show significant correlation with gestational age. A further critical step in resting-state fMRI analysis is the nuisance correction, where nuisance means both predictable and random sources of image noise. Here, the steps for MR image analysis were adapted for fetal functional imaging to ensure that maturing macroscopic-scale neural activity contributes to the observed signal intensity fluctuations. Although the current strategy is to obtain noise signals from areas where no neurons reside, the spurious effects of non-neuronal origin cannot be excluded, and, furthermore, monitoring of possible physiological noise sources would be desirable. We experienced a high exclusion rate of clinical cases on the grounds of excessive movement or other artifacts, greatly reducing the data available for analysis.

A possible limiting factor comes from the fact that our study relied on a manually defined ROI system. Localization in the fetal cortex is not directly possible by anatomical atlases, but rather, via the transfer of adult landmarks based on non-linear matching of geometry. This limits the spatial correspondence between subjects. It is possible that the propagation of cortical loci through similarity-based transformations introduces a bias toward later gestational ages when the convoluted surface is more ideal for localization and inter-subject correspondences are better. A possible solution to this problem is the use of functional data-driven parcellation of the cortex, such as demonstrated by Craddock et al. (2012) on the group level. It is possible to use group level ICA to reveal spatially coherent networks within the resting-state fMRI data. Although the transformation of our fetal fMRI data into one reference space for group ICA may suffer from interpolation artifacts due to the large volumetric and anatomical differences.

## OUTLOOK AND CONCLUSION

Understanding the formation of functioning neural ensembles in the human brain is particularly important for a model of healthy development, and such a functional, developmental model could be a standard to which pathological conditions can be compared. The assessment of the critical late gestational sculpting of fetal brain activity and macroscopic-scale functional circuitry is feasible with *in utero* neuroimaging. The optimization of imaging sequences and post-processing is crucial and must include strategies to address fetal head motion and non-neuronal sources of fMRI signal. The use of functional connectivity on a ROI basis will allow future investigations on disease development and its correlation to healthy characteristics of connectivity development curves, as shown in this work.

## AUTHOR CONTRIBUTIONS

András Jakab has written the manuscript, designed the experiments, performed the analysis and created figures. Ernst Schwartz has performed the analysis and contributed to the manuscript. Gerlinde M. Gruber has acquired data and contributed to the manuscript. Gregor Kasprian has written the manuscript and provided expert consultation. Daniela Prayer has acquired data, written manuscript and provided expert consultation, Veronika Schöpf, Georg Langs has written the manuscript, designed the experiments, provided senior supervision.

## Conflict of Interest Statement

The authors declare that the research was conducted in the absence of any commercial or financial relationships that could be construed as a potential conflict of interest.

## ABBREVIATIONS

BOLD: blood oxygen level dependent contrast
CSF: cerebrospinal fluid
fMRI: functional magnetic resonance imaging
FOV: field of view
FWHM: full width at half maximum
GW: gestational week
ICA: independent component analysis
MRI: magnetic resonance imaging
NBS: network-based statistics
ROI: region of interest
SSE: sum of squares for error
TE: echo time
TR: repetition time.
