# Supplementary material for: Fetal functional imaging portrays heterogeneous development of emerging human brain networks
Source: Front Hum Neurosci. 2014 Oct 22;8:852. doi: 10.3389/fnhum.2014.00852 (PMC4205819; doi:10.3389/fnhum.2014.00852)
Supplement: Supplementary file 1 [file Data_Sheet_1.DOCX]

**Signal intensity model for intrinsic functional connectivity in fetuses**

The localization of such non-neural signals is based on anatomical priors that can be adapted to the individual fetal brain through atlases or image segmentation. Here we present the adaptation of the CompCor method.

The grey matter (neuronal-) BOLD signal, *b_adult_*, during resting-state, task-negative experiments, is typically modeled as:

$$b_{adult}=A+S_{rs}+N_{p}+M+\varepsilon$$

with A a constant and linear term, S_rs_ contains the neuronal signal (resting-state activity), N_p_ is physiological noise, M are the motion confounds (caused by spin-history artifacts and the actual spatial voxel-displacement by intra-scanner head motion), and ε describes random additional noise. In the following paragraphs, we extend this formula by specifying the components of physiological noise and motion.

The motion confounds, M, can be modeled as frame-wise global displacements with six degrees of freedom:

$$b_{adult}=A+S_{rs}+N_{p}+M\left\{\begin{matrix} T_{x} \\ T_{y} \\ T_{z} \end{matrix}\begin{matrix} R_{x} \\ R_{y} \\ R_{z} \end{matrix}\right\}+\varepsilon$$

where T_x,y,z_: translations in the x, y, and z axis, and R_x,y,z_: rotations in x,y and z axis.

The physiological noise contributes substantially to the observed MRI signal, and, for the estimation of this component, a variety of algorithms exists. We utilized a script developed in-house, adopting the CompCor method, in which principal component analysis (PCA) separates the observed nuisance signal into individual time-courses. Such independently varying fluctuations of the MRI signal were reported to be consistent with the variability caused by respiratory and cardiac effects, and the approach was found particularly useful in de-noising fMRI data.

According to the CompCor model, three different aspects can be taken into consideration when locating the sources of noise signal: noise can be derived from image voxels comprising the brain ventricles and extra axial fluid spaces; white matter voxels; and voxels with the largest temporal variance. Thus, the previous formula can be extended to divide the noise signal into components from white matter, fluid spaces, and voxels with extreme temporal variability:

$$b_{adult,CompCor}=A+S_{rs}+N_{wmcsf}\left\{\begin{matrix} PC_{1} \\ PC_{2} \\ ... \\ PC_{N} \end{matrix}\right\}+N_{temp}\left\{\begin{matrix} PC_{1} \\ PC_{2} \\ ... \\ PC_{N} \end{matrix}\right\}+M\left\{\begin{matrix} T_{x} \\ T_{y} \\ T_{z} \end{matrix}\begin{matrix} R_{x} \\ R_{y} \\ R_{z} \end{matrix}\right\}+\varepsilon$$

where N_wmcsf_ anatomical noise sources (e.g., signals from white matter and cerebrospinal fluid voxels) are represented by the first N principal components; N_temp_ represents temporal noise, calculated by the voxels with the largest variance over the experiment.

In our experiments, we chose to adapt the method in (Behzadi et al., 2007), where the six strongest components are retained after the principal component analysis and the threshold for the noise voxels is in the highest two percentile on temporal standard deviation images.

White matter and CSF masks and were obtained from the segmentation of high-resolution fetal brain templates and were transformed to individual brains using the transformation fields from the spatial normalization step, as illustrated in Figure 5.

The spatial coverage of typical resting condition fMRI scans in fetuses involves ancillary structures and possible sources of confounding BOLD signals, such as the amniotic fluid, placenta, and maternal organs. Therefore, components of the surrounding regions are included in the model as explanatory variables:

$$b_{fetus,CompCor}=A+S_{rs}+N_{wmcsf}\left\{\begin{matrix} PC_{1} \\ PC_{2} \\ ... \\ PC_{N} \end{matrix}\right\}+N_{temp}\left\{\begin{matrix} PC_{1} \\ PC_{2} \\ ... \\ PC_{N} \end{matrix}\right\}+N_{external}\left\{\begin{matrix} PC_{1} \\ PC_{2} \\ ... \\ PC_{N} \end{matrix}\right\}+M\left\{\begin{matrix} T_{x} \\ T_{y} \\ T_{z} \end{matrix}\begin{matrix} R_{x} \\ R_{y} \\ R_{z} \end{matrix}\right\}+\varepsilon$$

where N_external_ are the principal components of the extra-fetal and extra-axial structures.

While studies on the functioning adult brain generally report that meaningful signals are coherent in the 0.001 – 0.1 Hz range (or a more strict limitation of 0.01 – 0.08 Hz), we did not have such established parameters for fetal data. Therefore, we defined the magnitude squared spectral coherence between each regional (filtered) fMRI time-series in each fetus and searched for a similar, low-frequency peak that is usually found in postnatal resting state fMRI experiments, or in adults.

**Testing fetal functional brain graphs against random networks and evaluating developmental trends**

In the next paragraph, we describe the procedure used to construct random brain graphs with architecture similar to that of the putative fetal networks. We sought to model the extent to which graph structure is influenced by the spatial adjacency of nodes in such a way that the correlation between their BOLD time-courses stems from shared, spurious, residual nuisance signals that spatially extend over many neighboring regions and are smoothed. For this, we relied on the following initial assumptions: (a) connections were considered non-random if their strengths were not explained by the variance of respective connections found in a set of simulated random brain graphs with similar node topology; (b) the probability that two nodes were falsely connected was higher if the two nodes were closer to each other, as their time-courses are influenced by the spurious effects of adjacent brain voxels; and (c) the relative position of the graph nodes do not change significantly during gestation, and thus, it is sufficient to determine only one matrix of pair-wise distances.

*D* is a randomized distance matrix for *N* brain regions (graph nodes) with a hypothesized spatial uncertainty of nodes due to inaccurate image registration:

$$\text{D}_{\text{i,j}=\text{1...N,i}\neq\text{j}}=\sqrt{(i_{x}\mu RND_{0...1}-j_{x}\mu RND_{0...1})^{2}+i_{y}\mu RND_{0...1}-j_{y}\mu RND_{0...1})^{2}+i_{z}\mu RND_{0...1}-j_{z}\mu RND_{0...1})^{2}}$$

where $\mu$ is a weighting factor controlling the spatial uncertainty allowed, *RND* are variables with random distribution, and *i_x,y,z_* and *j_x,y,z_* are the spatial coordinates of any given region pairs 1…N.

The adjacency (i.e., connectivity) matrix, *A,* of the simulated random brain graph is derived from an undirected, weighted random graph, with the strengths of connections weighted inversely with the Euclidian distance, assuming a Gaussian relationship between connection strength and nodal distance:

$$A_{\text{i,j}=\text{1...N,i}\neq\text{j}}=e^{-\frac{D_{\text{i,j}=\text{1...N,i}\neq\text{j}}^{2}}{2c^{2}}}RND_{\text{i,j}=\text{1...N,i}\neq\text{j}}\phi$$

The adjacency matrix is then normalized so that its largest element is 1:

$$A_{\text{i,j}=\text{1...N,i}\neq\text{j}}=A_{\text{i,j}=\text{1...N,i}\neq\text{j}}\frac{1}{\max_{\text{i,j}=\text{1...N,i}\neq\text{j}} A}$$

Where ${RND}_{i,j=1\ldots N,i\neq j}$ is an *N* by *N* matrix with random elements uniformly distributed between 0 and 1, *c* is the width of the Gaussian function so that $c=\frac{FWHM}{2\sqrt{2\ln2}}$where FWHM is the full width at half maximum of the filter function, *D* is the randomized distance matrix, and $\varphi$ is a weighting factor controlling the overall uncertainty of spurious connections.

A non-randomness test was performed for 32 fetal brain graphs using the NBS-based (Zalesky et al., 2010) linear model in which the topology was evaluated against 32 simulated random brain graphs. In these simulated graphs, the nodes’ spatial coordinates were set to the center of the mass points in the region system in the 37th gestational week. The parameter controlling the FWHM of the smoothing function was estimated from the actual data, by fitting a Gaussian function on the elements of the population-averaged matrix and the distance matrix, and variance caused by noise was set to 50% ($\varphi=0.5$). For the final graph analysis, only those connections were retained that were significantly different from the simulated random brain graphs.

References

Behzadi Y, Restom K, Liau J, Liu TT (2007): A component based noise correction method (CompCor) for BOLD and perfusion based fMRI. Neuroimage 37: 90-101.

Zalesky A, Fornito A, Bullmore ET (2010): Network-based statistic: identifying differences in brain networks. Neuroimage 53: 1197-1207.
